# Context Memory Encoding and Retrieval Temporal Dynamics are Modulated by Attention across the Adult Lifespan

**DOI:** 10.1523/ENEURO.0387-20.2020

**Published:** 2021-01-21

**Authors:** Soroush Mirjalili, Patrick Powell, Jonathan Strunk, Taylor James, Audrey Duarte

**Affiliations:** Department of Psychology, Georgia Institute of Technology, Atlanta, GA 30318

**Keywords:** aging, attention, context memory, episodic memory, multivariate pattern analyses

## Abstract

Episodic memories are multidimensional, including simple and complex features. How we successful encode and recover these features in time, whether these temporal dynamics are preserved across age, even under conditions of reduced memory performance, and the role of attention on these temporal dynamics is unknown. In the current study, we applied time-resolved multivariate decoding to oscillatory electroencephalography (EEG) in an adult lifespan sample to investigate the temporal order of successful encoding and recognition of simple and complex perceptual context features. At encoding, participants studied pictures of black and white objects presented with both color (low-level/simple) and scene (high-level/complex) context features and subsequently made context memory decisions for both features. Attentional demands were manipulated by having participants attend to the relationship between the object and either the color or scene while ignoring the other context feature. Consistent with hierarchical visual perception models, simple visual features (color) were successfully encoded earlier than were complex features (scenes). These features were successfully recognized in the reverse temporal order. Importantly, these temporal dynamics were both dependent on whether these context features were in the focus of one’s attention, and preserved across age, despite age-related context memory impairments. These novel results support the idea that episodic memories are encoded and retrieved successively, likely dependent on the input and output pathways of the medial temporal lobe (MTL), and attentional influences that bias activity within these pathways across age.

## Significance Statement

The events we learn and remember in our lives consist of simple context details like color and more complex ones like scenes. Whether we learn and recognize these memory details successively or simultaneously, and whether attending to some features but not others impacts when we encode and retrieve them is unknown. Using high temporal resolution neural activity patterns, we found color details were successfully encoded earlier than scene ones but recognized in the reverse order. Importantly, these temporal dynamics depended on which feature was in the focus of one’s attention and were preserved across age. These findings elucidate the successive manner in which the features that constitute our memories are encoded and retrieved and the conditions that impact these dynamics.

## Introduction

Numerous episodic memory studies have investigated the neural underpinnings of successful encoding and retrieval of different kinds of context features including color, spatial, and various semantic attributes (Uncapher et al., 2006; Awipi and Davachi, 2008; Duarte et al., 2011; Staresina et al., 2011; Park et al., 2014; Liang and Preston, 2017). Although several regions support successful episodic encoding and/or retrieval regardless of the nature of the context features, others are content-selective. Little is known about the time course with which different context features are successfully encoded and retrieved.

Why would the temporal dynamics of successful context encoding and/or retrieval be impacted by context feature type? Numerous perception studies have established that simple features like color are discriminated earlier in time and by earlier visual cortical regions than more complex features like scenes (Carlson et al., 2013; Kravitz et al., 2014; Clarke et al., 2015). Some regions supporting feature perception also support successful encoding of the features to which they are sensitive (Hayes et al., 2007; Awipi and Davachi, 2008; Preston et al., 2010; Dulas and Duarte, 2011). It is therefore possible that simple context features may be successfully encoded into memory before complex ones.

Context features may not be retrieved in the same order in which they are perceived. In one recent study researchers used multivariate pattern analyses (MVPAs) of electroencephalography (EEG) activity to decode the times at which perceptual and high-level conceptual information was discriminated and later reconstructed from memory (Linde-Domingo et al., 2019). Consistent with feed-forward visual processing hierarchies (Carlson et al., 2013; Kravitz et al., 2014), perceptual details were discriminated earlier than were more complex, conceptual ones. Interestingly, these temporal dynamics were reversed during recall. These results, together with intracranial EEG evidence showing reversed information flow within the medial temporal lobe (MTL) between encoding and retrieval (Fell et al., 2016), support the idea that remembering may proceed in reversed order from perception.

The reversal of information flow between perception and remembering is intriguing, but several questions remain. First, it stands to reason that simple features that are perceived earlier would also be successfully encoded into memory earlier than those perceived later. If complex features are reactivated earlier than simple ones (Linde-Domingo et al., 2019), one’s ability to successfully recognize a complex feature should also occur earlier. Second, normal aging is associated with neurocognitive slowing (Salthouse, 1996), with EEG and MEG studies showing processing delays for multiple neural components (Onofrj et al., 2001; Zanto et al., 2010; Clarke et al., 2015). Whether this slowing might also be observed for the time courses of simple and complex context feature encoding and/or retrieved is unknown. Third, in real world situations, one’s attention may be directed to the processing of some features over others. If attention is directed to high-level episodic features over low-level ones, for example, it is not clear that low-level features would be prioritized to the same extent during encoding. Indeed, ample evidence from event-related potential (ERP) studies of attention show earlier ERP latencies for attended than unattended visual stimuli (Hillyard and Anllo-Vento, 1998; Woodman, 2010).

**Figure 1.**
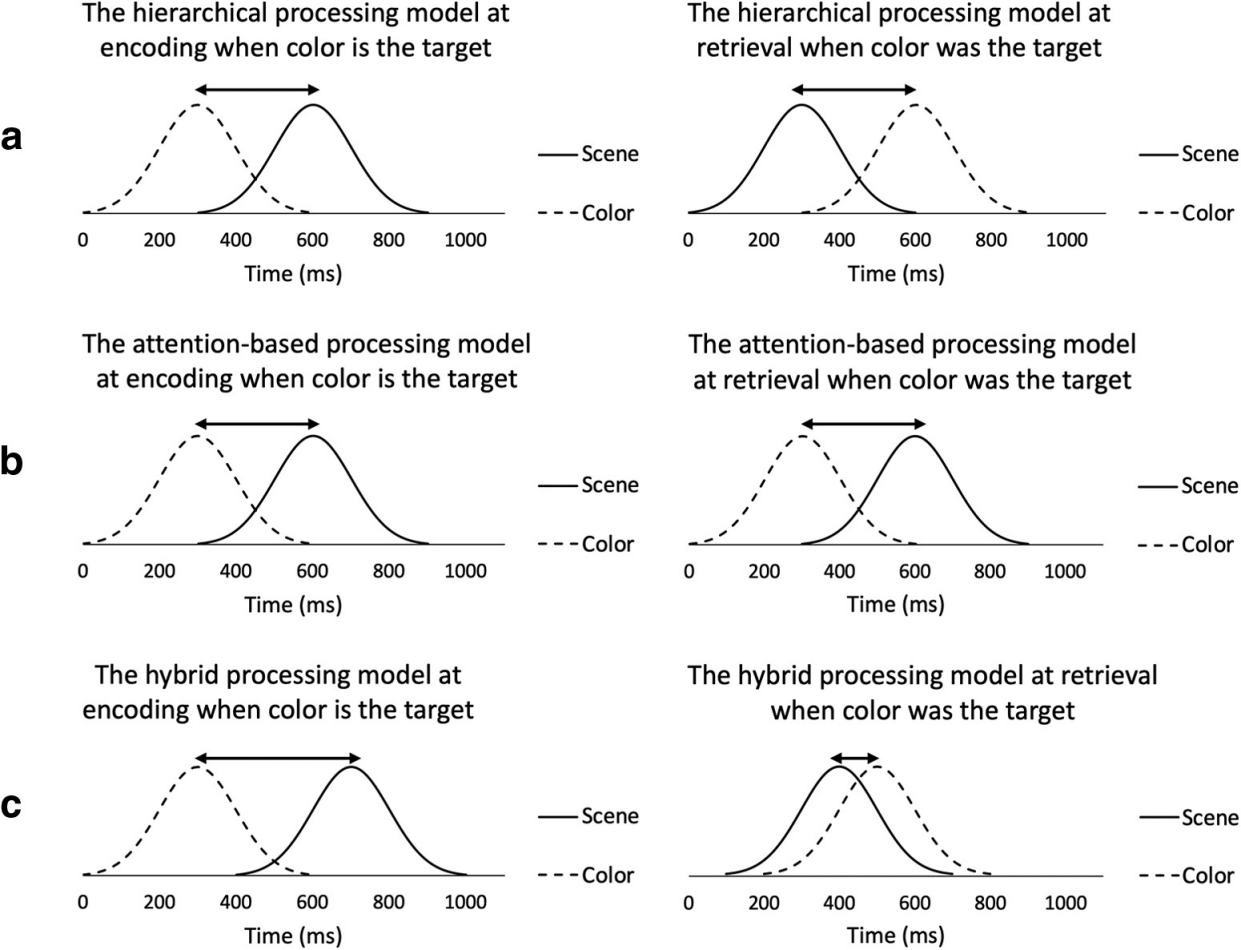
Three hypothesized model fits for low-order feature (color) and high-order feature (scene) context encoding and retrieval temporal dynamics. Predicted data for each model represent the earliest local classification peaks of context memory success decoding (correct vs incorrect) across the participants. In the hierarchical processing model (***a***), low-level context features, in this case color, processed by earlier visual cortical areas, are encoded before and retrieved following high-level ones, in this case scene, regardless of whether they were attended to during encoding. For this model, if scene were the target context, the encoding and retrieval histograms would be identical to those shown. Alternatively, in the attention-based processing model (***b***), the attended context feature will be encoded and retrieved earlier than the feature which is ignored. As shown in part ***b***, color encoding precedes scene encoding when color is the attended “target” feature, and the same temporal dynamic would hold at retrieval. If scene were the target, the order of the histograms would be reversed from those shown. Lastly, in the hybrid processing model (***c***), the temporal dynamics of encoding and retrieval are based both on the complexity of the context features, and whether they were the target or the distractor. In the example in part c, scene encoding follows color encoding by a longer delay when color is the target than when it is the distractor, while scene retrieval precedes color retrieval by a shorter delay. If scene were the target, the distance between the peaks would decrease for encoding and increase for retrieval compared with what is shown.

Here, we investigated the time courses of successful encoding and recognition of simple and complex perceptual features, and how attention might impact these temporal dynamics across the adult lifespan. Attentional demands were manipulated by having participants attend to the relationship between an object and either a color or scene while ignoring the other context feature. For both encoding and retrieval, we trained multivariate pattern classifiers to distinguish successful from unsuccessful context memory separately for color and scene features from oscillatory EEG. We assessed context memory classification accuracy through time for each feature as a function of whether or not they were attended to during encoding. We explored the fit of our data to one of three models (Fig. 1).

If the feed-forward processing hierarchy at encoding and reversed temporal dynamics at retrieval are unalterable, and independent of one’s current goals, we predict that results will fit the hierarchical model, across age. However, if attention modulates these dynamics, we predict that the fit to either the attention or hybrid models will be reduced with age; as the ability to selectively attend to task-relevant features is reduced with age (Hasher and Zacks, 1988; Campbell et al., 2010). Any attention modulation on the temporal dynamics should be reduced, potentially contributing to age-related context memory impairments (James et al., 2016a; Powell et al., 2018).

## Materials and Methods

### Participants

The participants consisted of 52, right-handed adults (21 women) from ages 18 to 74. Data from an additional five older adults (61–76 years) were excluded: two for lack of understanding of task procedures, two for noisy EEG (i.e., DC drift, movement), and one for computer malfunction. Data from one young adult (21 years) were excluded because of noisy EEG. A subset of the young and older, but not middle-aged, adults’ data were included in prior published studies examining different research questions (James et al., 2016b; Strunk et al., 2017; Powell et al., 2018). All subjects were native English speakers and had normal or corrected vision. Participants were compensated with course credit or $10/h and were recruited from the Georgia Institute of Technology and surrounding community. None of the participants reported any neurologic or psychiatric disorders, vascular disease, or use of any medications that impact the central nervous system. Participants completed a battery of standardized neuropsychological tests that consists of subtests from the memory assessment scale (Williams, 1991), including list learning, recognition, verbal span forward and backwards, immediate and delayed recall, visual recognition, recall, reproduction, and delayed recognition. Participants that scored >2 SDs outside the sample mean were excluded. Moreover, older adults were administered the Montreal cognitive assessment (MoCA; Nasreddine et al., 2005) to test further for mild cognitive impairments. Only participants scoring a 26 or above for the MoCA were included. All participants signed consent forms approved by the Georgia Institute of Technology Institutional Review Board.

### Materials

A total of 432 grayscale images of objects were selected from the Hemera Technologies Photo-Object DVDs and Google images. At encoding, 288 of these objects were presented; in half of the trials, participants’ attention was directed to a color and in the other half directed to a scene. Each grayscale object was presented in the center of the screen and a color square and scene were presented on the left or right of the object. For all trials in a block, the same context feature type was presented on the same side of the object. Piloting showed that this minimized participant confusion and eye movement artifacts. The locations of these context features were counterbalanced across blocks so that they were shown an equal number of times on the right-hand and left-hand side of the object in the center. For each encoding trial, participants were instructed to focus on associations between the object and either the colored square or the scene, which served as the target context for that trial. The potential scenes included a studio apartment, cityscape, or island. The scenes were taken from Creative Commons. The potential colored squares consisted of green, brown, or red. Each of the context and object pictures spanned a maximum vertical and horizontal visual angle of ∼3°. During retrieval, all 288 objects were included in the memory test in addition to 144 new object images that were not presented during encoding. Study and test items were counterbalanced across subjects.

### Experimental design and statistical analyses

**Figure 2.**
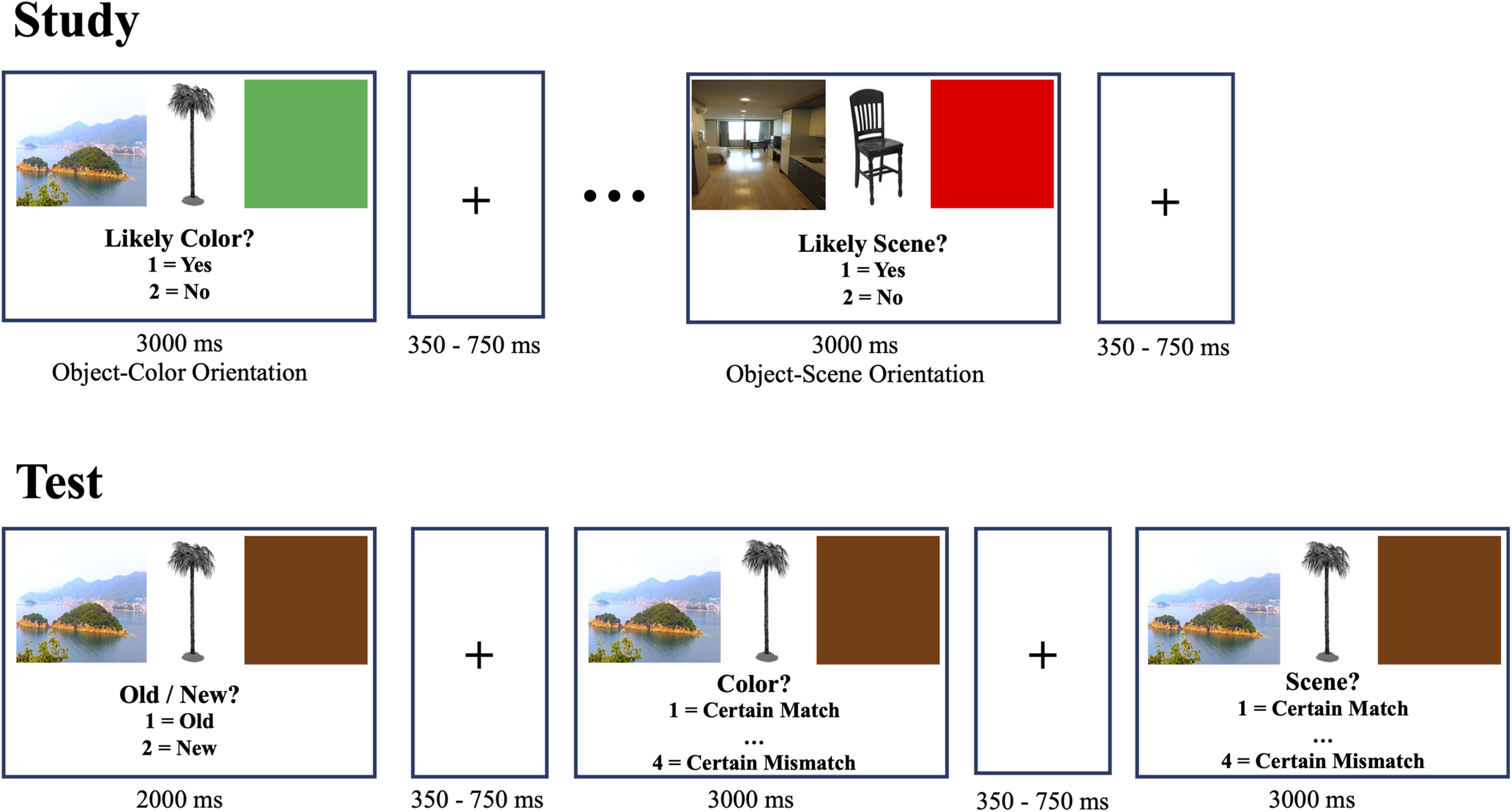
Experimental design. During study, participants were asked to make a subjective yes/no assessment about the relationship between the object and either the colored square (i.e., “is this color likely for this object?”), where one of three possible colors was presented (red, green, brown) or the scene (i.e., “is this object likely to appear in this scene?”), where one of three possible scenes was presented (cityscape, studio apartment, island). Participants were directed to pay attention to one context and ignore the other context. During test, participants made up to three responses for each test trial (item recognition, and color and scene context memory decisions).

Figure 2 illustrates the procedure used during the study and test stages. Before the beginning of each phase, participants were provided instructions and given 10 trials for practice. For the study stage, participants were asked to make a subjective yes/no assessment about the relationship between the object and either the colored square (i.e., “is this color likely for this object?”) or the scene (i.e., “is this object likely to appear in this scene?”). Instructions for the task specified that on any specific trial, the participant should pay attention to one context and ignore the other context. Within the study phase, there were four blocks where each block consisted of four mini blocks and each of them included 18 trials. In advance of beginning each mini-block, participants were provided a prompt (e.g., “Now you will assess how likely the color is for the object” or “Now you will assess how likely the scene is for the object”). Since prior evidence has suggested that memory performance in older adults is more disrupted when they have to switch between two distinct kinds of tasks (Kray and Lindenberger, 2000), mini blocks were used to orient the participant to which context they should pay attention to in the upcoming trials. Moreover, it decreases the task demands of having to switch from judging one context (e.g., color) to judging the other (e.g., scene). Each trial in a mini block had a reminder prompt presented below the pictures during study trials (Fig. 2).

During test, participants were presented with both old and new objects. Similar to the study phase, each object was flanked by both a scene and a colored square. For each object, the participant initially decided whether it was an old or a new image. If the participant detected the object was new, the next trial began after 2000 ms. If participants stated that it was old, then they were asked to make two additional assessments about each context feature and their certainty about their judgment (i.e., one about the colored square and another about the scene). The order of the second and third questions was counterbalanced across participants. For old items, the pairing was set so that an equal number of old objects were presented with: (1) both context images matching those presented at encoding stage, (2) only the color matching, (3) only the scene matching, and (4) neither context image matching. Responses to the context questions were made on a scale from 1 (certain match) to 4 (certain mismatch). There were four study and four test blocks. Young adults finished all four study blocks before the four test blocks. For older adults (over 60), to better equate item memory performance with young adults and to allow us to explore age effects in the EEG temporal dynamics unconfounded by large age effects in general memory ability (Rugg and Morcom, 2005), the memory load was halved so that they finished a two-block study-test cycle twice (two study, two test, two study, two test). All participants completed a short practice of both the study and test blocks before starting the first study block. Thus, participants knew of the upcoming memory test although they were not told to focus on their encoding decisions and not to memorize for the upcoming test.

#### Data collection

Continuous scalp-recorded EEG data were recorded from 32 Ag-AgCl electrodes using an ActiveTwo amplifier system (BioSemi). Electrode position is based on the extended 10–20 system (Nuwer et al., 1998). Electrode positions consisted of: AF3, AF4, FC1, FC2, FC5, FC6, FP1, FP2, F7, F3, Fz, F4, F8, C3, Cz, C4, CP1, CP2, CP5, CP6, P7, PO3, PO4, P3, Pz, P4, P8, T7, T8, O1, Oz, and O2. External left and right mastoid electrodes were used for referencing offline. Two additional electrodes recorded horizontal electrooculogram (HEOG) at the lateral canthi of the left and right eyes and two electrodes placed superior and inferior to the right eye recorded vertical EOG (VEOG). The sampling rate of EEG was 1024 Hz with 24-bit resolution without high or low pass filtering

#### EEG preprocessing

Offline analysis of the EEG data was performed in MATLAB 2015b using the EEGLAB, ERPLAB, and FIELDTRIP toolboxes. The continuous data were down sampled to 256 Hz, referenced to the average of the left and right mastoid electrodes, and band pass filtered between 0.5 and 125 Hz. The data were then epoched from –1000 ms before stimulus onset to 3000 ms. The time range of interest was from stimulus onset to 2000 ms, but a longer time interval is required to account for signal loss at both ends of the epoch during wavelet transformation. Each epoch was baseline corrected to the average of the whole epoch, and an automatic rejection process deleted epochs in which a blink occurred during stimulus onset or epochs with extreme voltage shifts that spanned across two or more electrodes. The automated rejection processes identified epochs with the following parameters in the raw data. (1) The voltage range was greater than 99th percentile of all epoch voltage ranges within a 400-ms time interval (shifting in 100-ms intervals across each epoch). (2) The linear trend slope was higher than the 95th percentile of all epoch ranges with a minimum $R^{2}$ value of 0.303) The voltage range was larger than 95th percentile of all epoch voltage ranges within a 100-ms time interval (shifting in 25-ms intervals across each epoch), between −150 and 150 ms from stimulus onset for frontal and eye electrodes only. Then an independent component analysis (ICA) was run on all head electrodes for identifying ocular artifacts (i.e., blinks and horizontal eye movements). Components related to ocular artifacts were omitted from the data by visually inspecting the topographic component maps and component time course with the ocular electrodes. Each epoch was re-baselined to the –300 to –100-ms time period before stimulus onset since the epochs were no longer baselined to a specific time period after deleting components related to ocular activity. If a dataset had a noisy electrode (e.g., >30% of the data required to be rejected), it was deleted from the processing stream and interpolated using the nearby channels to estimate the activity within the bad channel before running the time frequency procedure. After all processing stages, ∼13% (SD = 8%) of the epochs were removed.

#### Frequency decomposition

Each epoch was transformed into a time frequency representation using Morlet wavelets with 78 linearly spaced frequencies from 3 to 80 Hz, at five cycles. During the wavelet transformation, each epoch was decreased to the time interval of interest and down sampled to 50.25 Hz. For the following MVPAs, we examined only trials in which participants correctly recognized objects as old (item hits). The decision to select only item hit trials was based on the assumption that correct recognition of the associated contexts was contingent on correct recognition of the centrally-presented object. The average number of trials for younger, middle-aged, and older adults are as follows: younger (M = 190.50, SD = 41.01), middle-aged (M = 187.31, SD = 40.24), older (M = 177.06, SD = 38.56).

#### Time-resolved classification

We were interested in classifying the earliest time at which color and scene context features were successfully encoded and retrieved. In order to maximize the number of trials available to train the classifier, we collapsed across confidence levels for both correct and incorrect trial types at both encoding and retrieval. That is, some participants had very few trials for specific confidence conditions (e.g., correct context with high confidence) making it difficult to include confidence in classification analyses including all participants. Similarly, for retrieval, we collapsed across all trial types (i.e., both context images matching those presented at encoding stage, only the color matching, only the scene matching, and neither context images matching) to increase power to detect the effects of interest. It is important to note that the proportions of these trial types were roughly equivalent for context correct and incorrect trials (context correct: 29.5% both contexts match, 23.2% only color match, 22.1% only scene match, 25.2% neither context match; context incorrect: 20.7% both contexts match, 27.5% only color match, 28.0% only scene match, 23.8% neither context match). These proportions were roughly similar for the different attention conditions (i.e., attend color vs attend scene). For each classification analysis, we selected a specific 300-ms sliding time interval and shifted the time window by one time point (20 ms) over the initial 2-s period of the encoding and the item memory portion retrieval epochs (i.e., starting at stimulus onset at both encoding and retrieval). This 300-ms time interval was chosen to maximize information available for the classifier to separate correct from incorrect trials while also allowing for sufficient temporal resolution to detect peak latency differences between conditions. The first 2 s was chosen for classification analysis to be consistent with previous EEG studies, including ones using this same task, showing episodic memory effects within this time range (Rugg and Curran, 2007; James et al., 2016a; Powell et al., 2018). That is, even during the item recognition period, EEG activity is sensitive to context memory accuracy. Second, sampling of later time periods of the trial produced similar and/or less significant effects than those presented. Third, because the color and scene context recognition questions were presented and responded to later in the trial, we aimed to reduce the potential influence of color and scene perception on memory success effects. Subsequently, for each 300 ms interval, we extracted features based on common spatial patterns (CSPs) from the data at each frequency band separately, including δ (3–4 Hz), θ (4–7 Hz), α (8–14 Hz), β (14–30 Hz), and γ (30–80 Hz). The CSP algorithm aims to increase the discriminability by learning spatial filters which maximize the power of the filtered signal and minimize the power for the other class (Herbert et al., 2000). Briefly, the average covariance matrices of the trials of each class are computed, producing $\bar{C_{1}}$ and $\bar{C_{2}}$ for the two classes. Subsequently, using the concept of eigen value decomposition, an optimization problem of $w=arg\;\max\;\frac{w^{T}\bar{C_{1}}w}{w^{T}\bar{C_{2}}w}$ is solved to find the optimum spatial filters. In other words, the spatial filters optimally project the signals of the current space (i.e., across original electrodes) into a new space in which the signal at each projected electrode is a linear combination of the signals across all original electrodes and the variances of these signals is highly discriminable for the trials of the two classes (i.e., context correct vs context incorrect). Next, once the spatial filters across different frequency bands were extracted separately, we applied Fisher’s criteria to select the best features for each individual to reduce the feature space for training the classifier (Phan and Cichocki, 2010). To be consistent across all analyses and participants, and to avoid the risk of overfitting and underfitting based on the number of trials, we selected the best five features with the highest Fisher scores for each analysis. Finally, we trained a naive Bayesian classifier to distinguish the correct from incorrect context trials (Fukunaga, 1993). We used 5-fold cross-validation average accuracy as our criteria for evaluating the classifier’s performance. As a result, for each participant, we obtained one classifier accuracy value for each of the 86, 300-ms intervals (with the resolution of 20-ms sliding timepoints i.e., [0, 300 ms], [20, 320 ms], [40, 340 ms],…, [1700, 2000 ms]) for each phase of the experiment (encoding, retrieval), attention condition (target, distractor), and context feature (color, scene). While the theoretical chance level for binary classification problems is 50%, there are some studies that have shown the true level of chance performance can be remarkably different from the theoretical value (Combrisson and Jerbi, 2015; Jamalabadi et al., 2016). As a result, we used permutation tests (Nichols and Holmes, 2002) by repeating the classification analysis to obtain an empirical null distribution for the classifier performance. To be more specific, for each separate analysis and participant, we conducted the same time-resolved 5-fold cross-validation classification procedure as for the real data with true labels but used labels that were randomly shuffled at each repetition. This process was conducted 500 times per participant for each of the classification analysis with random label assignment on each repetition. This established an empirical null distribution of classification performance scores. Subsequently, we set the accuracy, which was higher than 95% of the performance values in the null distribution, as the threshold for determining the significance of a classifier’s performance for each subject. But it is important to note that each time interval will have its own empirical null distribution, and the 95th percentile for the null distribution is different across different time intervals, and to be more conservative, we have selected the highest 95th percentile across the time intervals as the threshold for that subject and analysis.

**Figure 3.**
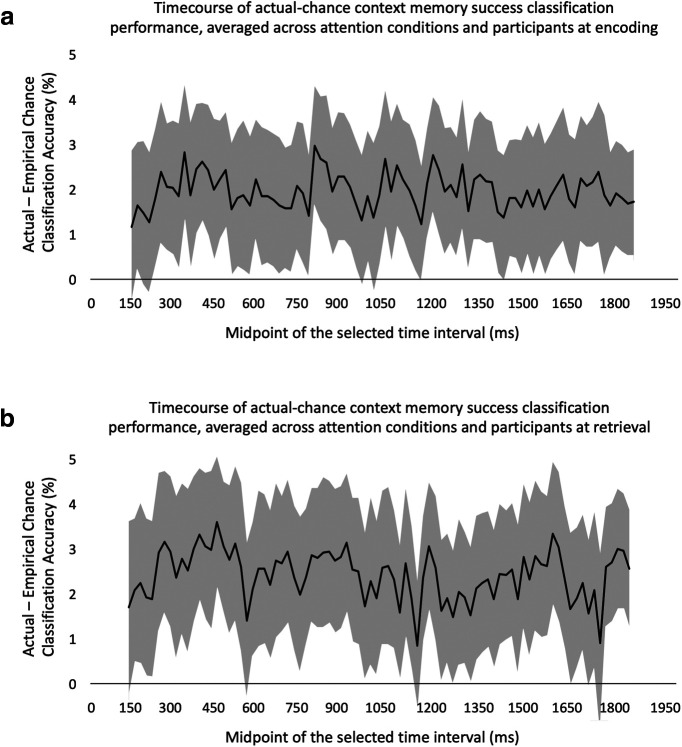
The time course of actual-chance context memory success classification performance, averaged across attention conditions and participants at (***a***) encoding and (***b***) retrieval with the 95% confidence intervals. Each time point in these diagrams represents the midpoint of the associated 300-ms time interval. Since the first time interval includes 0–300 ms, the diagrams start from 150 ms and end with 1850 ms, the midpoint of the last time interval (1700, 2000 ms). The gray area in each figure indicates the 95% confidence interval of the actual-chance context memory success classification performance across participants. If the gray area of a specific time point reaches 0%, the actual performance is not significantly different from chance, across participants, for the associated 300-ms time interval. For example, at encoding, the confidence interval associated with time point 1030 ms, the midpoint of the 880–1180 ms interval, reaches zero.

In order to show that classification performance is significantly above chance across subjects, and to show general time periods of memory success decodability through time, we subtracted the time course of each participant’s empirical chance level from the individual’s actual classification performance time course. We then averaged these difference time courses across the attend color and attend scene conditions. Finally, we averaged these individual difference time courses across participants. These across participant, average real-chance classification time courses for encoding and retrieval and 95% confidence intervals are shown in Figure 3. As can be seen in Figure 3, classification performance was significantly greater than chance, across subjects, for much of the encoding and retrieval time intervals. Context memory success was maximally decodable between 680–980 ms at encoding (midpoint of 830 ms; Fig. 3*a*) and between 340 and 640 ms at retrieval (midpoint of 490 ms; Fig. 3*b*).

**Figure 4.**
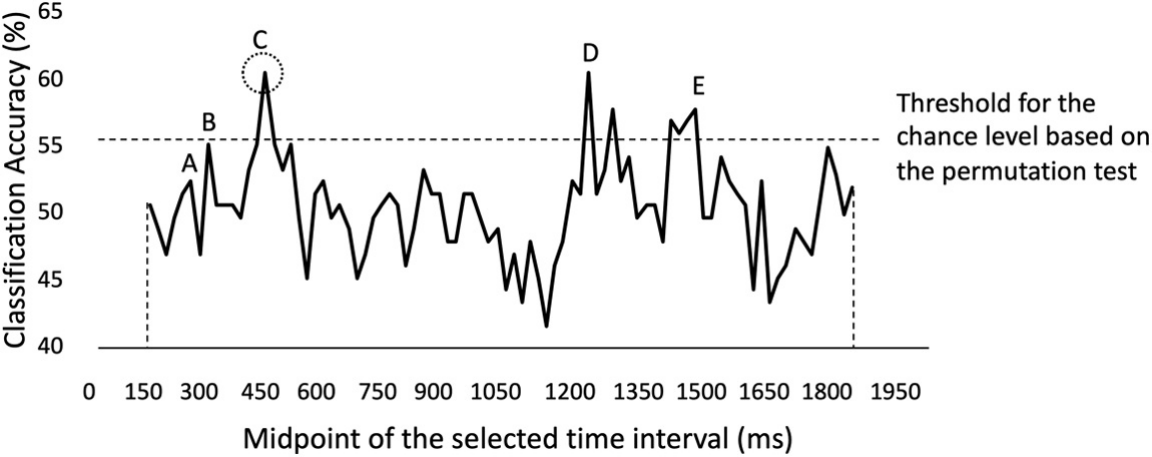
An example of the results of time-resolved context memory accuracy classification from a representative subject and classification analysis. Each time point in the diagram represents the midpoint of the associated 300-ms time interval. Since the first time interval includes 0–300 ms, the diagram starts from the midpoint of this time interval, as shown by the left vertical dashed line. Moreover, the diagram ends with the midpoint of the last time interval (1700, 2000 ms) as shown by the right vertical dashed line. Note that the threshold is set as the highest 95th percentile value through time in the time-resolved null distribution for each subject to be more conservative (see Materials and Methods).

Finally, we plotted the classifier accuracy values on a diagram where each point on the diagram (Fig. 4) represented the midpoint of each 300-ms time interval. In each of these diagrams, there would be multiple time intervals whose classification accuracy are higher than the adjacent time intervals (i.e., the time intervals right before and right after the current time interval, with 20-ms midpoint difference). However, since there would be many moments that qualify for this criterion, we expanded the adjacency interval to 60 ms. To be more specific, only the time intervals that had higher classification accuracy than all of the time intervals within their 60-ms temporal neighborhood were selected as the potential peak moments. For instance, in Figure 4, while A has higher performance than the time intervals right before and after, it cannot be selected as a potential peak since B is in its defined neighborhood and has higher performance. Moreover, the selected peak moments should perform significantly above the chance level. As a result, any potential peak moments that had lower performance than the significance threshold would not be considered. Again, in Figure 4, B will not be considered since it has performed less than the empirical chance level. Lastly, if there were multiple peaks that performed above the empirical chance level, the earliest would be selected as the “peak moment” that determined for the first time whether a context feature would be encoded/retrieved successfully. As can be seen in Figure 4, there are some peaks, including C, D, and E, that are qualified after both of the mentioned criteria, and we would select C as the peak moment in that particular analysis.

#### Code accessibility

The custom code that we used in this study is available from https://doi.org/10.17605/OSF.IO/FVUZX.

#### Data accessibility

The data and results that support the findings of this study are available from https://doi.org/10.17605/OSF.IO/FVUZX.

## Results

### Behavioral results

**Figure 5.**
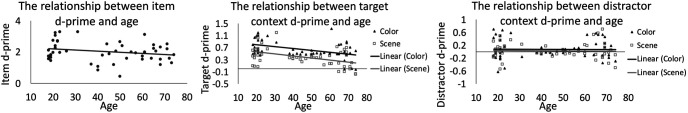
Item, color, and scene context memory discriminability.

We computed item memory *d′* to assess the effect of age on item recognition. To assess the effect of age on context memory performance, we computed *d′* for color and scene features when they were the target, and distractor, separately, using the following formula: *d′* = Z (proportion of “match” responses to contexts that matched those presented at encoding) – Z (proportion of match responses to contexts that mismatched those shown at encoding). Average item *d′* was 2.051 (SD* *= 0.626), and significantly above chance (0) (*t*_(51)_* *=* *23.623, *p *<* *0.0001). Age was not a significant predictor of item memory discriminability (*R*^2^ = 0.0335, *F*_(1,50)_ =1.73, β = −0.0056, *p *=* *0.194), indicating roughly stable item memory accuracy across age (Fig. 5).

One-sample *t* tests showed that while target *d′*, collapsed across color and scene, was significantly above chance (0), across participants (M* *=* *0.917, SD = 0.550, *t*_(51)_ = 12.028, *p *<* *0.001) distractor *d′* was not significantly above chance (M* *=* *0.048, SD = 0.214, *t*_(51)_ = 1.619, *p *=* *0.112). Furthermore, using paired sample *t* tests, as can be seen in Figure 5, memory discriminability was greater for targets than distractors (*t*_(51)_ = 11.162, *p *<* *0.001), and for color targets than scene targets (*t*_(51)_ = 3.934, *p *<* *0.001). There were no significant differences in *d′* for color and scene distractors (*t*_(51)_ = 1.005, *p *=* *0.320). Linear regression analyses confirmed that age was a significant negative predictor of *d′* for both color (*R*^2^ = 0.101, *F*_(1,50)_ = 5.63, *β* = −0.0104, *p* = 0.022) and scene (*R*^2^ = 0.175, *F*_(1,50)_ = 10.60, *β* = −0.0121, *p* = 0.002) targets but not distractors (color: *R*^2^ = 0.0004, *F*_(1,50)_ = 0.020, *β* = −0.0003, *p* = 0.888, scene: *R*^2^ = 0.0013, *F*_(1,50)_ = 0.065, *β* = −0.0004, *p* = 0.80).

We investigated the response times based on the context memory judgements at encoding and retrieval. For retrieval, the subjects had to make three different decisions if they identified the item as old, we were interested in the average response times of their item recognition decisions as a parallel to the EEG analyses. For each participant, we computed an average of the response times for the attend color and scene context memory conditions and for context correct and incorrect trials during encoding and retrieval. The average response time for each across participants is shown in Table 1.

**Table 1:** Average response times for encoding and retrieval based on context memory success for each context feature

	Encoding		Retrieval	
Correct	Incorrect	Correct	Incorrect	
Color	1354.5 ms (147.0)	1377.3 ms (153.8)	1356.8 ms (241.8)	1400.2 ms (273.3)
Scene	1356.5 ms (166.9)	1379.8 ms (178.4)	1361.2 ms (215.2)	1357.6 ms (208.0)

The associated SDs are reported in parentheses.

ANOVAs conducted for encoding and retrieval periods showed that effects of context (color, scene), accuracy (correct, incorrect), and the interaction were all non-significant (encoding, context: *F*_(1,204)_ = 0.01, *p* = 0.922; accuracy: *F*_(1,204)_ = 1.05, *p* = 0.306; interaction: *F*_(1,204)_ < 0.01, *p* = 0.990; retrieval, context: *F*_(1,204)_ = 0.37, *p* = 0.542; accuracy: *F*_(1,204)_ = 0.34; *p* = 0.563, interaction: *F*_(1,204)_ = 0.48, *p* = 0.491). The lack of significant differences suggests that classifier performance patterns described below were not likely influenced by response time (i.e., motor activity) differences between conditions.

### EEG results

#### Evidence for the hybrid model: attention modulates the temporal dynamics of successful context feature encoding and retrieval, across age

As stated previously, we posited three potential models to describe the temporal dynamics of encoding and retrieval of low-order and high-order visual context features (as shown in Fig. 1). In the hierarchical model, the temporal dynamics are based on complexity alone, independent of the feature that is the focus of attention. The attention-based model is determined solely by the focus of attention. The hybrid model is a combination of these two. In order to determine the fit of our data to these models, we conducted several time-resolved Bayesian classification analyses (see Materials and Methods) to obtain a temporal map of discriminability between correct and incorrect trial types for each context feature (color, scene), attention condition (target, distractor), subject, and memory phase (encoding, retrieval). Classifier performance was assessed using a 300-ms sliding time window, slid in 20-ms increments across the entire 2000-ms encoding or retrieval epoch such that 86 classifier performance values were obtained. An illustrative example of the results of time-resolved context memory decoding which is obtained from one condition (i.e., classification of color corrects vs incorrect at encoding when color was the target) for one participant is shown in Figure 4. We present this exemplar of a single analysis for one participant, instead of an average of time-resolved performance across participants because the threshold of the empirical chance level is different for each analysis and participant (see Materials and Methods). In Figure 4, each time point represents the midpoint of the associated 300-ms time interval. Point C is the highest local peak among its neighbors (<60-ms distance) and would be selected as the earliest local peak for context memory decoding for subsequent analyses.

**Figure 6.**
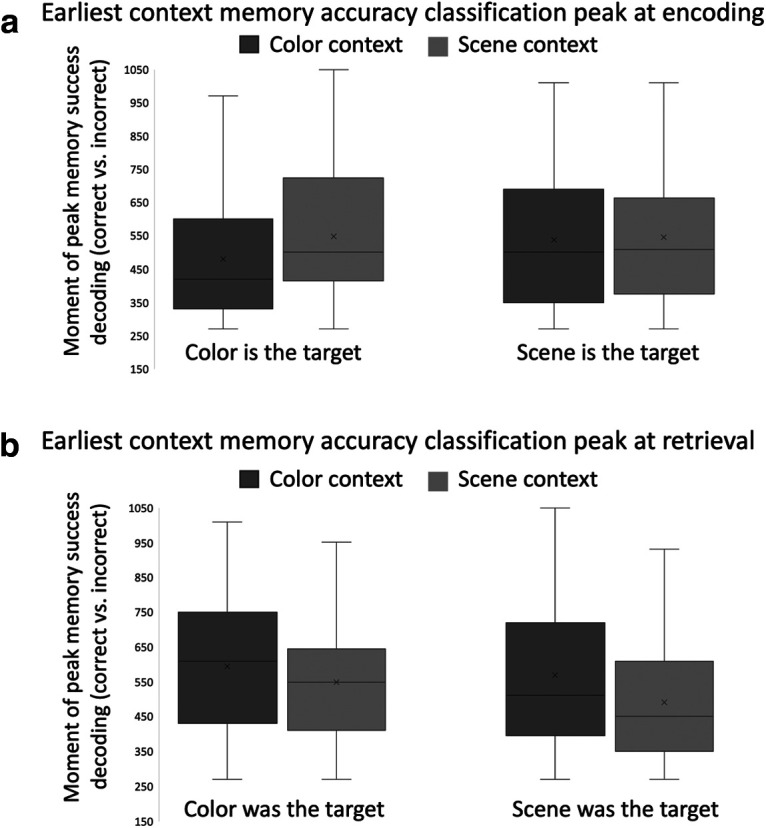
The earliest peaks of the context memory accuracy (correct vs incorrect) classification averaged across participants at (***a***) encoding and (***b***) retrieval. At each stage of the experiment, we divided the trials based on the target at encoding. We performed a set of MVPAs to discriminate context (i.e., color or scene) correct and incorrect trials for each attention condition and memory phase; × indicates the condition means while the horizontal lines indicate the medians.

For each participant, there were eight local peaks selected: color context memory decoding when color is the target; color context memory decoding when scene is the target; scene context memory decoding when color is the target; scene context memory decoding when scene is the target, for each memory phase (encoding, retrieval). The earliest local classifier peaks that were significantly above chance level were selected for each separate participant for each context feature, attention condition, and memory phase and plotted in Figure 6.

Initially, we performed one-sample Kolmogorov–Smirnov tests (Marsaglia et al., 2003) to determine whether these local peaks followed a normal distribution. As the data were not normally distributed (Kolmogorov–Smirnov stat = 0.1045, *p *<* *0.001), we used one-tailed Wilcoxon sign-rank tests to test our predictions regarding the temporal dynamics of encoding and retrieval. We found that when color was the attended target feature, color was encoded significantly earlier than was scene (T* *=* *790.5, *p *=* *0.008). However, when scene was the target feature, the difference between the classifier peaks for color and scene was not significant (T* *=* *658.5, *p *=* *0.325), inconsistent with the hierarchical and attention models. A one-tailed Wilcoxon sign-rank comparison further confirmed that the time difference between peaks for color and scene encoding was significantly greater when color was the target context than when scene was the target (T* *=* *843, *p *=* *0.046). Finally, the peak for the target color preceded that for the target scene (T* *=* *852, *p *=* *0.039), inconsistent with the attentional model.

We conducted the same analysis of classifier peaks of color and scene context retrieval. When scene was the target feature during encoding, scene context was retrieved significantly earlier than was color across participants (T* *=* *884.0, *p *=* *0.019). By contrast, when color was the target feature during encoding, the difference between the classifier peaks for color and scene was not significant (T* *=* *806.0, *p *=* *0.091), inconsistent with the hierarchical and attention models. The direct comparison between scene and color peaks for the two attention conditions (i.e., scene was the encoding target vs color was encoding target) was not significant (T* *=* *749, *p *=* *0.211). Finally, the peak for scene retrieval preceded that for color even when each was the previously attended target context during encoding (T* *=* *934, *p *<* *0.001), inconsistent with the attentional model.

Collectively, these results suggest that when color is the target, attention and visual complexity are synergistic, and color features are encoded before scene features. However, when scene is the target, color and scene contexts are encoded at roughly the same time. Interestingly, the focus of attention during encoding also impacted the temporal dynamics at retrieval, albeit to a lesser degree than at encoding. Scenes are retrieved before color context features when previously attended but this latency effect is somewhat reduced and not significant when scenes were distractors. Results from both encoding and retrieval are most consistent with the hybrid model.

**Figure 7.**
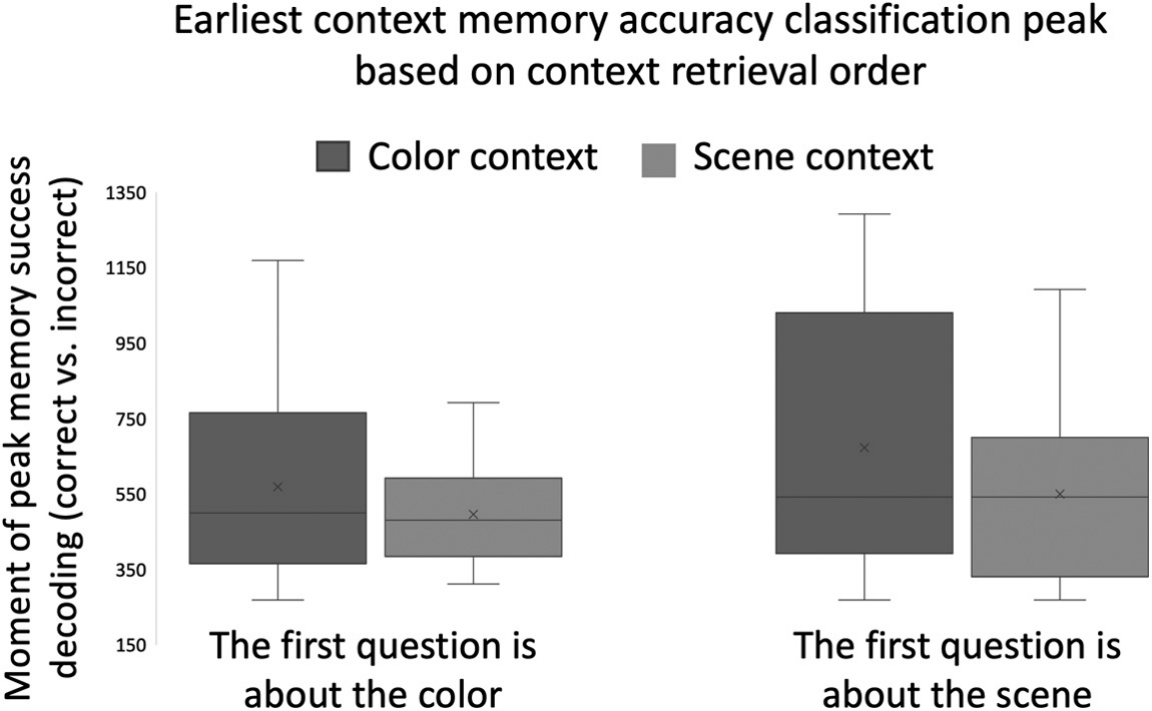
The earliest peaks of the context memory accuracy (correct vs incorrect) classification analyses for color and scene contexts, collapsed across encoding condition, compared between groups making either scene or color judgments first. As for the attention manipulation during encoding (Fig. 6), this pattern shows that one’s attentional state during retrieval impacts the temporal order of context memory retrieval.; × indicate the condition means while the horizontal lines indicate the medians.

In addition to the attention manipulation during encoding, there was an additional attention manipulation during retrieval in that some subjects were first asked to make scene context memory decisions before color decisions, while other subjects were asked to make color context memory decisions before scene decisions. The order of the questions was counterbalanced across participants. We investigated whether this between-subject factor impacted the temporal dynamics of context retrieval. For this analysis, we collapsed across encoding condition to increase the number of trials for classifier training. As seen in Figure 7, one-tailed Wilcoxon sign-rank tests showed that scene contexts were retrieved significantly earlier than color for participants that made scene context decisions before color decisions (T* *=* *162, *p *=* *0.017). This latency effect was reduced for participants that made color context decisions before scene decisions (T* *=* *245.5, *p *=* *0.089). As for the results described above, the focus of one’s attention at retrieval impacts the temporal dynamics of context memory retrieval, consistent with the hybrid model.

In order to assess any effect of age on these temporal dynamics, we wanted to determine whether there were any age-related slowing effects in the context memory decoding peaks. To this end, we ran a set of linear regressions to see whether age predicted the peaks for any of the conditions of interest (i.e., scene, color, encoding, retrieval). In none of these analyses was age a significant predictor of peak time (all $R^{2}s\;<\;$0.01, *F*s* *<* *1.723, *p*s* *>* *0.216), confirming no significant age-related slowing of context memory classification peaks. Next, we ran a series of linear regressions entering age as the predictor and the peak difference between color and scene for each attention condition and at both encoding and retrieval. In none of these regressions was age a significant predictor of the time difference (all $R^{2}s\;<\;$0.041, *F*s* *<* *2.120, *p*s* *>* *0.152). These null results support the idea that the processing hierarchy during encoding and reversal during retrieval and the impact of attention on these dynamics is preserved with age.

Finally, the context memory success classifiers, trained to distinguish correct from incorrect context memory trials, indicated that color context features are successfully encoded before scene context features and recognized in the opposite order during retrieval. However, they do not necessarily reveal that one context feature is processed/reactivated before the other. We conducted an additional classification analysis to explore this possibility. Specifically, we conducted time-resolved three-class classification analyses to identify the earliest peak time at which the presented colors and scenes were discriminated/processed during encoding and retrieval. We trained separate classifiers to discriminate colors (green, red, or brown) and scenes (studio apartment, cityscape, or island) and assessed their performance for the trials where the color or scene context was correctly remembered. The earliest local classification performance peaks that were significantly above chance were selected for each participant using the approach described above for the memory success classification. The earliest local classifier peaks that were significantly above chance level were selected for each separate participant for each context feature, attention condition, and memory phase. The mean peak moments of classification performance for each condition, averaged across participants, with the associated confidence intervals are shown in Table 2.

**Table 2.** Earliest peak moments of context feature decoding according to the context, attention condition, and memory phase averaged across participants

	Encoding		Retrieval	
	Color context decoding	Scene context decoding	Color context decoding	Scene context decoding
Color was target	427.8 (406.3, 449.3)	463.0 (435.4, 490.6)	463.5 (428.9, 498.1)	462.6 (429.9, 495.3)
Scene was target	442.6 (422.1, 463.1)	471.3 (451.9, 490.7)	447.0 (414.3, 479.7)	433.5 (415.1, 451.9)

95% confidence intervals are reported in parentheses (all of the reported values are in milliseconds).

Using a one-tailed Wilcoxon sign-rank test, we found that at encoding, when color was the attended feature, colors were discriminated earlier than were scenes (T* *=* *634, *p *=* *0.026). When scene was the target, while the peak moment for decoding colors preceded that for scenes, this difference was not significant (T* *=* *573, *p *=* *0.114). By contrast, at retrieval, when scene was previously the attended context feature during encoding, the peak moment for decoding scenes significantly preceded that for colors (T* *=* *442, *p *=* *0.044). Interestingly, when color was the previous target, the difference between the classifier peaks for color and scene decoding was not significant (T* *=* *425, *p *=* *0.216). Collectively, these feature discriminability results parallel those from the context memory accuracy classification analyses in support of the hybrid model. Specifically, colors are processed before scenes during encoding particularly when they are also in the focus of one’s attention. Scenes are discriminated before colors during retrieval but only when scenes were previously attended during encoding.

## Discussion

Events we experience are multidimensional, including simple, low-level context details such as color or shape, and more abstract or complex dimensions, such as spatial configural or conceptual abstractions. Vision neuroscience research shows that these dimensions are perceived hierarchically, from simple to complex (Carlson et al., 2013; Kravitz et al., 2014; Clarke et al., 2015), and as emerging memory research suggests, reconstructed in the reverse order (Linde-Domingo et al., 2019). It seems plausible, although yet untested, that simple context features would also be successfully encoded into memory earlier than those perceived later. Likewise, whether complex context features are successfully recognized before simple ones is unknown. Furthermore, no study has investigated the impact of attention or age on these temporal dynamics during encoding and retrieval. In this study, by capitalizing on the high temporal resolution of EEG signals recorded during episodic encoding and retrieval and applying MVPAs in an adult lifespan sample, we found that low-level perceptual context features (color) are successfully encoded earlier than high-level ones (scenes), and recognized in the reverse order during retrieval. Moreover, these temporal dynamics are dependent on attention both during initial learning and subsequent retrieval, such that these latency differences are robust only when the prioritized context feature (color during encoding and scene during retrieval) is also the focus of one’s attention. Finally, these temporal dynamics are robust across age, even in the presence of memory impairment.

As is typical in healthy aging studies (Naveh-Benjamin et al., 2007; Mitchell and Johnson, 2009), context memory accuracy was disproportionately impaired with age relative to item recognition, which was spared. For older adult participants over the age of 60, we halved the memory load (see Materials and Methods), allowing us to explore age effects in the EEG temporal dynamics unconfounded by large age effects in general memory ability (Rugg and Morcom, 2005). Across age, participants showed greater context memory discriminability for previously attended (target) than unattended (distractor) features, for both color and scene contexts. This pattern builds on previous work showing young and older adults alike can direct their attention toward task-relevant context details, while ignoring distractors, in a way that supports context memory performance (Glisky and Kong, 2008; Glisky et al., 2001; Dulas and Duarte, 2013). However, this attention manipulation cannot fully ameliorate age-related context memory impairments. As discussed below, the time-resolved decoding analyses are consistent with these behavioral results.

By applying MVPA-based classification of oscillatory EEG signals, we were able to identify the specific timepoints showing earliest decodability of successful versus unsuccessful encoding and retrieval of color and scene context features for each participant. During encoding, color context features were successfully encoded roughly 70 ms earlier than were scenes. By contrast, these temporal dynamics were reversed during retrieval by roughly the same latency. Although the poor spatial resolution of EEG precludes our ability to explore the neural generators of these activity patterns, existing memory theories suggest that retrieval cues facilitate recovery of elements of encoding episodes via the hippocampus (Eichenbaum, 2004). fMRI studies showing reinstatement of encoding-related activity during retrieval in the MTL and other stimulus-sensitive perceptual processing regions (Johnson et al., 2009; Kuhl et al., 2011; Staresina et al., 2012; Bosch et al., 2014; Wang et al., 2016; Bone et al., 2020), support this idea but the temporal sequence of these effects is unknown. If episodic retrieval proceeds in the reversed sequence as perception, then information supported by processing in regions nearer the hippocampus, like parahippocampal cortex, should be successfully recovered before information processed in more distal, extrastriate cortical areas (Morita et al., 2004; Aminoff et al., 2013). Our results showing that scenes were successfully recognized before colors is perfectly in line with this hypothesis. Future studies using methods with higher spatial resolution, including high-density scalp or intracranial EEG, could assess the relative timing of encoding and retrieval activity within these stimulus-sensitive brain regions.

It is important to note that the classifiers used to assess the aforementioned encoding and retrieval patterns were trained to distinguish successful from unsuccessful context memory decisions. These results clearly show that color context features are successfully encoded before scene context features and recognized in the reverse order, across age. However, these results do not necessarily suggest that color features were perceived before scenes during encoding and reactivated following scenes during retrieval. For example, temporal differences between memory success classification peaks might have also been observed if context memory decisions were easier to make for one feature than the other. However, the lack of reaction time differences between color and scene context memory decisions at either encoding or retrieval is inconsistent with this explanation. With regard to our study design, one might imagine that presenting scene and color context features during retrieval might have induced a more “perception-like” or “re-encoding” pattern, with color classification peaks preceding those for scene. As this was not the case, we believe that the influence of a retrieval mode in which task demands biased subjects toward memory recovery (Rugg and Wilding, 2000), rather than perception, and consequently, high-order perceptual features were recognized before low-level ones. The fact that this latency shift was most evident when scene features were previously attended and more well integrated with the objects in memory, as discussed below, further support this idea. Finally, we conducted separate classification analyses to determine the earliest peak time at which color and scene features could be successfully discriminated. The results from these analyses also showed that while colors were discriminated earlier than were scenes during encoding, the opposite temporal pattern was observed during retrieval. Collectively, we believe that our context memory success effects are best explained in terms of a feed-forward perception hierarchy and reversal of this hierarchy during remembering.

By manipulating participants’ attention, we could assess the extent to which the aforementioned temporal dynamics are a fixed feature of episodic memory or alternatively, fully or partially dependent on one’s attentional state. During encoding, the classifier peak for color context encoding significantly preceded that for scene only when participants attended to color. During retrieval, the classifier peak for scene context retrieval preceded that for color only when participants were first oriented toward scene context memory discriminations. These findings show that top-down attention impacts the degree to which low-level perceptual features are encoded before and retrieved after high-level ones. Importantly, the temporal dynamics were not fully dependent on attention, as the classification peak for the non-prioritized context (scene at encoding, color at retrieval) never preceded that for the prioritized feature even when it was the focus of attention. These results are consistent with an extensive neuroscience of attention literature showing that top-down attentional control biases activity within early sensory cortical areas as well as higher-order category-selective regions (Desimone and Duncan, 1995; Serences and Yantis, 2006; Gazzaley and Nobre, 2012). This bias manifests as both enhanced amplitude and reduced latency of activity associated with to-be perceived/encoded stimuli or those sought after in memory.

Interestingly, top-down attention during encoding also impacted the temporal dynamics of retrieval. Specifically, scene significantly preceded color context retrieval when scenes, but not colors, were previously attended to during encoding. What could explain this pattern of results? The attention manipulation during encoding, i.e., directing participants to specific object-context relationships, likely facilitated stronger bindings that were in turn more easily recovered during retrieval. More specifically, demands on strategic retrieval operations such as “postretrieval monitoring” are reduced when sought after information is easier to recover (Wilding and Rugg, 1997; Senkfor and Van Petten, 1998; Wilding, 1999; Kuo and Van Petten, 2006; Cruse and Wilding, 2009; Dulas and Duarte, 2013). Indeed, the time range encompassing the context memory decoding peaks overlaps that in which postretrieval monitoring ERPs are typically reported (∼600–1000 ms; Wilding and Rugg, 1996; Senkfor and Van Petten, 1998; Friedman and Johnson, 2000; Cruse and Wilding, 2009). If memory strength were the sole driver of the timing differences between classification peaks, then color context retrieval would have preceded scene context retrieval when color was the previously attended feature. Furthermore, color context *d′* exceeded scene *d′* but still, color context retrieval never preceded that for scene. Collectively, these memory success decoding patterns are most consistent with the idea that visual episodic features are retrieved in a reversed order from that in which they are encoded. Importantly, the degree to which these temporal dynamics are evident depends on one’s attention toward and mnemonic strength for the prioritized feature.

As aging has been shown to reduce one’s ability to selectively attend to task-relevant features in the presence of distractors (Hasher and Zacks, 1988; Campbell et al., 2010), we had predicted that any attention modulation on the temporal dynamics of encoding and retrieval might be reduced with age. However, attention-related modulations of context encoding and retrieval classification peaks were preserved across age. What might explain this? First, there were no age-related delays in the classification peaks for either context encoding or retrieval. While some previous EEG studies have shown age-related slowing of episodic memory-related ERPs (Trott et al., 1997, 1999; Mark and Rugg, 1998; Wegesin et al., 2002; Duarte et al., 2006; Swick et al., 2006; Gutchess et al., 2007) and oscillatory EEG effects (Strunk et al., 2017), others have not (Dulas et al., 2011; Newsome et al., 2012), and none have assessed latencies of mnemonic classification performance. It does not follow that age-related slowing, per se, would impact the hierarchical order of context feature encoding and retrieval. However, it is conceivable that if slowing were particularly evident for one feature, the difference between classification peaks might have been obfuscated. Second, although age effects were apparent in memory discriminability, target contexts were remembered better than were distractors, across age, suggesting that even older adults showed relatively strong selective attention and context memory performance. Thus, it is perhaps unsurprising that attention modulations of the temporal dynamics were unaffected by age. Reducing the memory load for older participants likely contributed to their generally strong memory performance. These results are generally consistent with previous aging studies showing that under conditions of relatively strong performance, patterns of neural activity contributing to episodic encoding and retrieval are roughly stable across age (Rugg and Morcom, 2005; Duverne et al., 2009; Angel et al., 2013; Chastelaine et al., 2015, 2016a,b).

Our results offer important insight into the temporally-dynamic nature of context encoding and retrieval across the adult lifespan. Consistent with feed-forward visual perception hierarchical models, simple features such as colors are encoded before more complex, scene features, with a reversal of this order during retrieval. Importantly, these temporal dynamics are dependent on whether these features are in the focus of one’s attention. These results support the idea that episodic memories are created and recovered in a successive manner dependent both on the neuroanatomical pathways of the MTL and visual cortex, and top-down influences that bias activity within these pathways. Even in the presence of age-related episodic memory impairments, these dynamics are preserved with age. An interesting future question would be to understand whether neuropathology, as in Alzheimer’s disease, that greatly impacts the integrity of these neural pathways alters not only episodic memory ability but also its dynamic quality.

## Synthesis

Reviewing Editor: Alfonso Araque, University of Minnesota Decisions are customarily a result of the Reviewing Editor and the peer reviewers coming together and discussing their recommendations until a consensus is reached. When revisions are invited, a fact-based synthesis statement explaining their decision and outlining what is needed to prepare a revision will be listed below. The following reviewer(s) agreed to reveal their identity: Karen Campbell. Note: If this manuscript was transferred from JNeurosci and a decision was made to accept the manuscript without peer review, a brief statement to this effect will instead be what is listed below.

Your manuscript has been reviewed by the two original reviewers of the initial J Neurosci version.

Both reviewers have found merit and interest on the findings reported. I concur with the reviewers’ comments expressing the interest of the study.

Moreover, while they indicated that many comments on the previous version have been addressed, they also expressed some remaining concerns about several issues that need to be addressed and clarified. I also consider pertinent the concerns expressed.

Reviewers and I believe that the comments require less than two months of work.

I am looking forward to receiving a revised version of the manuscript that addresses the reviewer´s comments.

Sincerely,

Alfonso Araque

Reviewing Editor

eNeuro

Specific comments of the reviewers:

Reviewer 1.

This is a manuscript that I reviewed before (@JNeurosci). The study uses multivariate analyses of EEG patterns to investigate the dynamics of encoding and retrieving contextual information, and their modulation by attention and age. The results suggest that successful encoding of color context precedes the encoding of scene context information, and this hierarchy is reversed during old/new recognition. The findings are certainly of interest to the memory and ageing community. Many of my previous comemnts (summarized below with same numbering as original review) are now addressed. A number of concerns remain, however, and should be addressed in a further revision.

(1) Is a classifier trained on memory success well suited to reveal the time course of context feature reinstatement? The authors should clarify their approach and its implications.

-> In response to this comment, the authors now clarify, throughout the manuscript, that classifiers are trained on memory success for the main analyses. They also added more direct evidence from classifiers trained on context features, and the results support the central interpretation. This concern is thus sufficiently addressed.

(2) The authors should clarify what what portions of the recognition trials were used for the recognition-based classification.

-> The authors clarified that the old/new recognition portion of the trial was used for classification, sufficiently addressing this comment.

(3) The Methods section needs to explain how exactly the feature vectors were formed, and explain the CSP method.

-> The CSP method is now briefly outlined in the methods section. I am still unsure whether CSP was applied to each frequency band separately, and if the “optimal” feature vector in each subject was then a sub-selection of electrodes, concatenated across frequency bands. Also, it should be made explicit exactly how Fisher’s criterium was used to select the best feature (i.e., if there was a cut-off value).

(4) The Methods section needs to explain how the null distribution was generated, i.e. how many permutations, shuffling on single-subject level?

-> This comment was adequately addressed.

(5) The manuscript should have a figure showing the average classification performance over time for the encoding and retrieval data, with confidence intervals generated from the null distributions. Also, while the “illustrative example” in Fig. 4 is helpful for understanding the rationale behind peak selection, a sample of real data from a single subject would help the reader get an idea of how smooth the single-subject classifier time courses were in reality. The relatively long 300ms sliding time window for classification suggests that the time courses should in reality not be very peaky.

-> These comments have yet to be addressed in a revision.

(6) The manuscript should report button press latencies and their interaction with attention.

-> This analysis was added to the manuscript, and a table included to show the results. For retrieval, the average latency of item recognition for incorrect scene trials seems to be much longer (almost 700ms) than all other RTs. It would be good if the authors could include a measure of variance (SD or SEM) in the table to confirm that the lack of a significant interaction is due to large variance in response times.

(7) The results section should also describe (in the text or a figure) if significant decoding peaks came from all types of retrieval trials equally, or were unevenly distributed across both-match, color-match, scene-match and none-match trials.

-> I did not see this comment addressed in the revised manuscript. This is an important analysis to check the degree to which decodability of the context features was disproportionately driven by trials on which the correct color/scene features were visually presented, or by trials where the features were mentally reinstated.

One additional minor comment:

- Figure 5 should state what panels (a) and (b) refer to. I assume that (a) is encoding, (b) is retrieval, but this info is missing from the legend.

Reviewer 2.

In this paper, the authors use EEG and a source memory task to examine the temporal order of encoding and retrieval of simple (i.e., color) and complex (i.e., scene) contextual features and age differences therein. Using multivariate pattern analysis (MVPA) to predict correctly recognized versus incorrect trials, they show that simple visual features appear to be successfully encoded before complex features, and retrieved in the reverse order, but attention also seems to moderate these effects (in that attending to scenes, for instance, brings scene processing forward in time). Finally, these temporal order effects were not affected by age.

I reviewed this manuscript when it was previously submitted to the Journal of Neuroscience and highlighted a number of concerns in that review. The authors have done a good job of addressing most of my previous concerns. Most notably, I took issue with some of the wording/interpretation, in that a classifier trained to distinguish between correct/incorrect trials cannot tell you whether perceptual details are processed or perceived before more complex details (because the outcome measure reflects subsequent memory not current processing). The authors have now changed their wording throughout and included an additional analysis designed to test which context feature is being processed/reactivated (page 26).

In my view, the manuscript has been greatly improved and should be published pending some minor revisions. The findings are interesting and increase our understanding of the flow of information during encoding and retrieval, suggesting that this flow can be altered by top-down attentional goals. My few remaining concerns are detailed below.

1. For some of the analyses, it was still unclear which portion of the trial at retrieval was being analyzed (i.e., the item memory portion of the trial or the context retrieval part of the trial). Maybe this could be made clearer on page 14 - when you say “stimulus onset", which onset do you mean at retrieval?

2. In the results (p26 top), the authors should start a new paragraph at “Finally, the context memory success classifiers...”

3. The following sentence (on p26) does not make sense in isolation : “The mean peak moments of each condition across participants with the associated confidence intervals.”

4. Figure 5 - both captions are the same (i.e., no indication of which is encoding or retrieval).

5. Typo on p30: “might have induced a more “perception-like” or “re-encoding” pattern during, with color classification” - “during” should be removed?

## Author Response

Dear Dr. Araque:

Enclosed please find our revised manuscript entitled “Context memory encoding and retrieval temporal dynamics are modulated by attention across the adult lifespan.” Please consider it for publication as a research article in eNeuro.

We were pleased that both reviewers found our revision to be much improved (i.e. Reviewer #2 “In my view, the manuscript has been greatly improved and should be published pending some minor revisions. The findings are interesting and increase our understanding of the flow of information during encoding and retrieval, suggesting that this flow can be altered by top-down attentional goals.”). The reviewers had some remaining concerns and we have addressed them. We include our point-by-point response to the reviews (in quotes) for our initial submission to eNeuro.

Reviewer #1

(3) “The Methods section needs to explain how exactly the feature vectors were formed, and explain the CSP method. -> The CSP method is now briefly outlined in the methods section. I am still unsure whether CSP was applied to each frequency band separately, and if the “optimal” feature vector in each subject was then a sub-selection of electrodes, concatenated across frequency bands. Also, it should be made explicit exactly how Fisher’s criterium was used to select the best feature (i.e., if there was a cut-off value).”

We now make it explicitly in the Methods on pg. 15 that CSP was applied to each frequency band separately “Subsequently, for each 300 ms interval, we extracted features based on common spatial patterns (CSP) from the data at each frequency band separately, including delta (3-4 Hz), theta (4-7 Hz), alpha (8-14 Hz), beta (14-30 Hz), and gamma (30-80 Hz).”And just to emphasize this further, we restate this in the Methods on pg. 15 as well “Next, once the spatial filters across different frequency bands were extracted separately, we applied Fisher’s criteria to select the best features for each individual”.

Regarding the feature vectors, we have described that the CSP filter project the signals across electrodes into a new space of electrodes where the signals of the two classes are as discriminable as possible across the new electrodes. Moreover, the projected signal on each new electrode is a linear combination of the signals across all electrodes. we now make this explicitly in the Methods on pg. 15 “In other words, the spatial filters optimally project the signals of the current space (i.e., across original electrodes) into a new space in which the signal at each projected electrode is a linear combination of the signals across all original electrodes and the variances of these signals is highly discriminable for the trials of the two classes (i.e., context correct vs context incorrect).”

Lastly, since the CSP-based features for each subject and analysis received different sets of Fisher scores, it was not possible to set a unique cut-off value for the Fisher score for all of the analyses and participants. Instead, for each subject and analysis, we sorted the features based on their Fisher scores and selected the top 5 features for each classification analysis to be consistent across every analysis. Selecting top 5 features is a hyper-parameter of our classification model and it is set in order to avoid the risk of underfitting and overfitting the model. we now make this explicitly in the Methods on pg. 16 “To be consistent across all analyses and participants, and to avoid the risk of overfitting and underfitting based on the number of trials, we selected the best 5 features with the highest Fisher scores for each analysis.”

(5) The manuscript should have a figure showing the average classification performance over time for the encoding and retrieval data, with confidence intervals generated from the null distributions. Also, while the “illustrative example” in Fig. 4 is helpful for understanding the rationale behind peak selection, a sample of real data from a single subject would help the reader get an idea of how smooth the single-subject classifier time courses were in reality. The relatively long 300ms sliding time window for classification suggests that the time courses should in reality not be very peaky. -> These comments have yet to be addressed in a revision.

While we definitely could add this figure to the manuscript, we came to the conclusion that this figure might not be very informative since each subject and analysis has its own empirical chance level and showing the average chance level along with the average classifier performance through time could be misleading. That is, we think such a figure could make it unclear to the reader that the peaks were detected in an individualized manner. The average threshold for chance across participants is 58.2% and the average classification performance across time intervals is above average chance level for most of the time intervals and is smoother compared to a temporal diagram of a single participant. For your reference, this is how that figure looks like:

We now state this explicitly in the Results on pg. 21 that we could add this figure but we decided not to do so since it wouldn’t add much to the manuscript “We present this exemplar of a single analysis for one participant, instead of an average of time-resolved performance across participants because the threshold of the empirical chance level is different for each analysis and participant (see Method).”

Moreover, the diagram in Figure 4 reflects actual data from one participant and we now make this explicitly in the Results on pg. 20 “An illustrative example of the results of time-resolved context memory decoding which is obtained from one condition (i.e., classification of color corrects vs incorrect at encoding when color was the target) for one participant is shown in Figure 4.”

(6) The manuscript should report button press latencies and their interaction with attention. -> This analysis was added to the manuscript, and a table included to show the results. For retrieval, the average latency of item recognition for incorrect scene trials seems to be much longer (almost 700ms) than all other RTs. It would be good if the authors could include a measure of variance (SD or SEM) in the table to confirm that the lack of a significant interaction is due to large variance in response times.

We have added the standard deviations for the response times for each condition in Table 1 in the Results on pg. 19.

(7) The results section should also describe (in the text or a figure) if significant decoding peaks came from all types of retrieval trials equally, or were unevenly distributed across both-match, color-match, scene-match and none-match trials. -> I did not see this comment addressed in the revised manuscript. This is an important analysis to check the degree to which decodability of the context features was disproportionately driven by trials on which the correct color/scene features were visually presented, or by trials where the features were mentally reinstated.

First of all, we performed an additional behavioral analysis to investigate how evenly the context correct and incorrect trials come from the 4 types of retrieval trials (both-match, color-match, scene-match and none-match). We have added this in the Methods on Pg. 14 “It’s important to note that the proportions of the these trial types were roughly equivalent for context correct and incorrect trials [context correct: 29.5% both contexts match; 23.2% only color match; 22.1% only scene match; 25.2% neither context match), (context incorrect: 20.7% both contexts match; 27.5% only color match; 28.0% only scene match; 23.8% neither context match)]. These proportions were roughly similar for the different attention conditions (i.e., attend color vs attend scene).”

Moreover, we could not break the available trials into 4 different sets of trials based on types of retrieval trials (both-match, color-match, scene-match and none-match) since the number of trials for each classification analysis would decrease significantly to a degree that it wouldn’t be possible to properly train the classifier. As a result, the time-resolved classification performances was obtained by using all the available trials and it was not possible to break each diagram (for an analysis of a subject) into 4 additional diagrams based on types of retrieval trials and find 4 sets of additional peak values as well for each person and analysis.

One additional minor comment: - Figure 5 should state what panels (a) and (b) refer to. I assume that (a) is encoding, (b) is retrieval, but this info is missing from the legend.

This is now corrected in Figure 5.

Reviewer #2

1. For some of the analyses, it was still unclear which portion of the trial at retrieval was being analyzed (i.e., the item memory portion of the trial or the context retrieval part of the trial). Maybe this could be made clearer on page 14 - when you say “stimulus onset", which onset do you mean at retrieval?

We now make this explicitly clear in the Methods on pg. 14 “we selected a specific 300 ms sliding time interval and shifted the time window by one time point (20 ms) over the initial 2 second period of the encoding and the item memory portion retrieval epochs (i.e. starting at stimulus onset at both encoding and retrieval).”

2. In the results (p26 top), the authors should start a new paragraph at “Finally, the context memory success classifiers...”

This is now corrected.

3. The following sentence (on p26) does not make sense in isolation : “The mean peak moments of each condition across participants with the associated confidence intervals.”

This is now corrected in pg. 27 “The mean peak moments of classification performance for each condition, averaged across participants, with the associated confidence intervals are shown in Table 2.”

4. Figure 5 - both captions are the same (i.e., no indication of which is encoding or retrieval).

This is now corrected in Figure 5.

5. Typo on p30: “might have induced a more “perception-like” or “re-encoding” pattern during, with color classification” - “during” should be removed?

This is now corrected.

We hope that our revised manuscript is acceptable for publication in eNeuro. Thank you for your consideration.

Sincerely,

Soroush Mirjalili, B.S.
